# Supplementary material for: Patterns of Intron Gain and Loss in Fungi
Source: PLoS Biol. 2004 Nov 30;2(12):e422. doi: 10.1371/journal.pbio.0020422 (PMC532390; doi:10.1371/journal.pbio.0020422)
Supplement: Table S1 — Also available at http://genes.mit.edu/NielsenEtAl/. (4.3 MB ZIP). [file pbio.0020422.st001.zip › NielsenEtAl/html/1395.html]

AN2316.1.NCU05457.1.MG03188.1.FG04315.1


```
 CLUSTAL W (1.82) Multiple Sequence Alignments - Introns Inserted


Sequence 1: MG03188.1	167 aa
Sequence 2: FG04315.1	174 aa
Sequence 3: NCU05457.1	171 aa
Sequence 4: AN2316.1	197 aa
Alignment Length: 202 aa
Number Identitical Residues: 71 aa
Alignment Score (without introns) 3750


MG03188.1 	MLRT-TGSAMLRRSVVQAT-----------------------------ARRASSTHAISK
NCU05457.1	MLRTPTVSALVRNVAVRAAKP-------------------------TMAVRAASTMPISN
FG04315.1 	MLRT-SATNLLRKSLVRSTP--------------------------ALASRAASTHAISN
AN2316.1  	MFAPSISRAVARSSAMPMPAIRSYRTISSPMTCVNARPQAEKKSIAAQQTRAASEHAIAN
          	*: ..    : *   :  .   :  : ::. :. .: ..:...: ::   **:*  .*::

MG03188.1 	PTLANIEKRWESMPLQEQAELWMSLRERMKENWNELTLHEKKAA1YWVSFGPHGPRALDP
NCU05457.1	PTLANIEKRWEQMPMQEQAELWMALRDRMKGNWADLTLQEKKAA1YYIAFGPHGPRALPP
FG04315.1 	PTLANIEKRWEGMPLQEQAELWMALRDRMQSNWTELTLQEKKAA1YWIAFGPHGPRAEDP
AN2316.1  	PTLAGIEKRWEALPPQEQADLWMQLRDRMKVDWHQMTLQEKKAA1YWIAFGPHGPRSQPP
          	****.****** :* ****:*** **:**: :* ::**:***** *:::*******:  *

MG03188.1 	PDEGKKVAMGVAIGLAVSFMIFATMRSFAKPAPHTMTKEWQEQTNEYLK0-----SQNTE
NCU05457.1	PGEQKKVLAYTVAGVFLSFVIFATMRAFAKPPPATMTKEWQEATNEFLK0-----AQKSD
FG04315.1 	PGTNARVAWGIFIGIAASVGLFGLVRLAAKPAPYTMTQEYQEETNEFLK0LTRTQNQKSD
AN2316.1  	KGENLRIFFKVAQLCVVSVGLFYFIHLFAKPLPKTMSKEWQEATNEYAL0-----REKIN
          	 .   ::          *. :*  ::  *** * **::*:** ***:         :: :

MG03188.1 	PLTGLSSEGYSGKGQVQSPPASKH
NCU05457.1	PLTGLTSEGYNGKGHVQSPSASA-
FG04315.1 	PFTGITSPGYAGKGMVQSPPKGN-
AN2316.1  	PIYGVSSQGYEGKGFVQSPPADKS
          	*: *::* ** *** ****. .
```
